# Acral Lentiginous Melanoma With NRAS Mutation and Ocular Surface Toxicity Following Immunotherapy and Investigational KRASG12C Inhibitor: Case Report

**DOI:** 10.1155/crom/7529882

**Published:** 2026-04-09

**Authors:** Elisah Huynh, Andrew Gregory, Peter Y. Chang

**Affiliations:** ^1^ The Ocular Immunology and Uveitis Foundation, Waltham, Massachusetts, USA; ^2^ Massachusetts Eye Research and Surgery Institution, Waltham, Massachusetts, USA, mersi.us; ^3^ University of Massachusetts Chan School of Medicine, Worcester, Massachusetts, USA

**Keywords:** anti-RAS, corneal epithelial defect, daraxonrasib, metastatic melanoma

## Abstract

We report the case of a patient with metastatic foot melanoma enrolled in a clinical trial of the anti‐RAS agent RMC‐6236 who developed chronic bilateral corneal epithelial defects and thinning, likely secondary to the systemic effects of targeted therapy. This case highlights the ocular surface toxicity associated with systemic anticancer therapies affecting rapidly dividing cells and the overall importance of multidisciplinary medical management of these systemic therapeutics.

## 1. Introduction

Acral lentiginous melanoma (ALM) is a rare and aggressive subtype of cutaneous malignant melanoma typically found on the palms, soles, and digits of the hands and feet. Although ALM represents only 2%–3% of all melanoma cases, it is associated with a poorer prognosis due to its aggressive behavior and tendency to be diagnosed at a more advanced stage [1, 2]. Factors in ALM that portend a poor prognosis, specifically survival, include older age, Breslow depth >2 mm, presence of ulceration at the tumor site, among many others [2].

Ocular side effects—such as corneal surface changes, conjunctivitis, and retinal toxicity—have been reported with the use of systemic chemotherapy agents [3, 4]. Therapies such as cetuximab, a monoclonal antibody that targets epidermal growth factor receptors, have been linked to corneal toxicity [5]. Targeted therapies for melanomas associated with NRAS mutation are a relatively recent development and remain active in clinical trials. Commonly reported side effects of this therapy include rash, nausea, diarrhea, and vomiting [6]. Although ocular side effects are a recognized complication of systemic chemotherapy, to our knowledge, there has not yet been a case reported in relation to the investigational agent RMC‐6236 [3, 4]. Here, we present a patient with metastatic ALM originating in the left foot, who was found to have persistent bilateral corneal erosions a few months after the initiation of RMC‐6236, a KRASG12C inhibitor (daraxonrasib).

## 2. Case Report/Case Presentation

### 2.1. Oncology History

A 55‐year‐old male presented in October 2022 with a persistent bleeding lesion on the left heel. A MRI revealed a 1.5‐cm enhancing dermal/subcutaneous lesion. Biopsy also confirmed a 7.5‐mm ALM (Clark V, 18 mitoses/mm^2^, pT4a, nonulcerated). Immunohistochemistry (IHC) was positive for SOX10 and MART‐1; molecular analysis identified an *NRAS* c.181C > A (p.Q61K) mutation. Staging PET/CT and brain MRI were negative.

The patient began adjuvant nivolumab (480 mg every 4 weeks) in May 2023. After nine cycles, CT imaging 7 months later revealed a new 8‐mm subcutaneous nodule at the posterior calcaneus, concerning for a recurrence. Biopsy of nodule on the medial and posterior part of his foot/ankle confirmed melanoma in dermis; positive for MART1, SOX10, PAME immunostains highlight lesional cells. In January 2024, he was switched to combination immunotherapy with ipilimumab (3 mg/kg) and nivolumab (1 mg/kg every 2 weeks).

Over the course of four cycles of ipilmumab and nivolumab, CT scan of the leg from April 2024 showed a new nodule appearing at the posterior calcaneus. Treatment options were discussed given the progression, and patient consented to Protocol 22‐297 of NRAS inhibitor in patients with NRAS mutation. Brain MRI was negative.

In April 2024, the patient enrolled in a Phase I/II trial (Protocol 22‐297, Dana‐Farber) investigating RMC‐6236, a KRASG12C inhibitor (daraxonrasib), at 300 mg daily. Side effects included nausea, vomiting, rash, mucositis, diarrhea, fatigue, electrolyte imbalances, and thrombocytopenia. Medication was held May 2024 due to side effects then resumed 1 month later with dose reduction at 200 mg daily. The medication was then held an additional time from August 2024 to October 2024 due to skin side effects.

As of June 2025, the patient continues on daraxonrasib and shows stable disease per RECIST guidelines (Response Evaluation Criteria in Solid Tumors) and partial response per iRECIST (immune resist used to evaluate tumor response to chemotherapy) [7]. He continues active follow‐up with oncology.

### 2.2. Ophthalmic History and Clinical Course

Four months after starting daraxonrasib, the patient presented to our tertiary eye care center August 2024 with bilateral blurry vision and photophobia. Visual acuity (VA) was right eye (OD) 20/40 (pinhole to 20/25), left eye (OS) 20/25–1. Slit‐lamp exam showed evidence of severe dry eye disease and a 2 × 3 mm nasal corneal abrasion OS. A bandage contact lens (BCL) was placed OS with moxifloxacin 0.5% TID OS and artificial tears initiated.

In the period of daraxonasib cessation September 2024, patient was noted to have healing corneal ulcer. However, after resuming chemotherapy, 1 month later, the patient was noted to have a new nasal corneal abrasion with haziness OS. He was treated with hourly moxiflocaxin, and a BCL was placed the following week.

Over the following months, he developed bilateral nasal epitheliopathy, more severe OS noted in November 2024 with stromal thinning of 60% OS and 30% OD by April 2025. No corneal ulcers or infiltrates were noted. VA fluctuated from 20/25 to 20/70 OS over these months.

Management included intermittent BCLs, topical moxifloxacin (TID OU), and preservative‐free artificial tears (QID OU). Oral vitamin C (1000 mg TID), loratadine, warm compresses, and lid hygiene were started to help with dry eye syndrome, allergic conjunctivitis, and corneal defects. Srecycline 100‐mg BID was added in to help with collagen defects.

Despite treatment, epithelial defects persisted with improved comfort. As of June 2025, VA was OD 20/40+ and OS 20/50–2 (improved to 20/30 with pinhole). OD had a stable 2‐mm nasal epitheliopathy with 30% thinning; OS showed persistent 3.25‐mm nasal neovascularization with 60% thinning.

## 3. Discussion

Corneal epithelial toxicity is a rare but important side effect of anticancer therapies, particularly those targeting rapidly proliferating cells [8]. RMC‐6236 is a small molecule inhibitor that targets the RAS signaling cascade in the MAPK pathway [9]. The inhibitor works by binding to active‐state RAS to form an inhibitory tricomplex, which turns off or disrupts the downstream activity of this pathway [10]. The MAPK pathway within cellular biology is fundamental in cellular development, differentiation, proliferation, and death. Dysregulations or mutations within this biological pathway are often found in human tumors and cancers [11].

The disruptions in both the normal mitotic and tumor cells could be the reason why this patient exhibited chronic, nonhealing epithelial defects and stromal thinning localized nasally in both eyes after starting the medication. This pattern suggests a localized area of limbal stem cell susceptibility or toxic accumulation of systemic drug or its metabolites, although further studies are needed.

To date, cases of MEK inhibitors within the MAPK pathway have been linked to retinal disorders, including chorioretinopathy and serous retinal detachment [9, 12]. Mild to severe ophthalmic toxicities described as visual disturbances, dry eye, and retinal toxicities have been reported [7]. However, there have been no case reports on the upstream small molecule inhibitor RAS in clinical trial RMC‐6236 reporting ocular surface toxicity to our knowledge.

Management of corneal defects is supportive including aggressive lubrication, antibiotic prophylaxis with topical moxifloxacin, and mechanical support via BCLs. High‐dose vitamin C was administered to support collagen synthesis and wound healing.

Importantly, the patient′s ocular toxicity mirrored the timing of systemic therapy exposure, with improvement during drug holidays and deterioration upon resumption. This temporal relationship supports the likely drug‐related etiology, although further investigation is required.

## 4. Conclusion

Patients receiving systemic targeted therapies, such as KRASG12C inhibitor, should be monitored carefully, as they may develop significant ocular surface complications. Awareness and early ophthalmic referral can prevent vision‐threatening symptoms. Further research is required to study the effects of KRASG12C inhibitors.

## Funding

No funding was received for this manuscript.

## Conflicts of Interest

Dr. Peter Chang: Co‐owner of MERSI; Speaker honoraria from Bausch & Lomb, Alimera Sciences, Alcon and Mallinckrodt Pharmaceuticals; Independent Contractor with Alimera, Mallinckrodt Pharmaceuticals, Genentech. Data & Safety Monitoring Board Chair for Ocugen.

## Data Availability

The data that support the findings of this study are available on reasonable request from the corresponding author.
